# Sulfur Fertilization Changes the Community Structure of Rice Root-, and Soil- Associated Bacteria

**DOI:** 10.1264/jsme2.ME15170

**Published:** 2016-03-05

**Authors:** Sachiko Masuda, Zhihua Bao, Takashi Okubo, Kazuhiro Sasaki, Seishi Ikeda, Ryo Shinoda, Mizue Anda, Ryuji Kondo, Yumi Mori, Kiwamu Minamisawa

**Affiliations:** 1 Graduate School of Life Sciences, Tohoku UniversityKatahira, Aoba-ku, Sendai, Miyagi 980–8577Japan; 2 National Institute for Agro-Environmental SciencesKannondai, Tsukuba, Ibaraki 305–8604Japan; 3 Department of Marine Bioscience, Fukui Prefectural UniversityObama, Fukui 917–0003Japan

**Keywords:** *Bradyrhizobiaceae*, paddy rice, sulfur oxidation, thiosulfate

## Abstract

Under paddy field conditions, biological sulfur oxidation occurs in the oxidized surface soil layer and rhizosphere, in which oxygen leaks from the aerenchyma system of rice plants. In the present study, we examined community shifts in sulfur-oxidizing bacteria associated with the oxidized surface soil layer and rice roots under different sulfur fertilization conditions based on the 16S ribosomal RNA (rRNA) gene in order to explore the existence of oligotrophic sulfur-oxidizing bacteria in the paddy rice ecosystem. Rice plants were grown in pots with no fertilization (control) or CaCO_3_ or CaSO_4_ fertilization. A principal-coordinates analysis (PCoA) showed that CaSO_4_ fertilization markedly affected bacterial communities associated with rice roots and soil, whereas no significant differences were observed in plant growth among the fertilizer treatments examined. In rice roots, the relative abundance of *Acidobacteria*, *Alphaproteobacteria*, *Gammaproteobacteria*, and TM7 was significantly higher in CaSO_4_-fertilized pots than in control pots. *Alphaproteobacteria*, *Bradyrhizobiaceae*, and *Methylocystaceae* members were significantly more abundant in CaSO_4_-fertilized roots than in control roots. On the other hand, the abundance of *Actinobacteria* and *Proteobacteria* was lower in CaSO_4_-fertilized soil than in control soil. These results indicate that the bacteria associated with rice roots and soil responded to the sulfur amendment, suggesting that more diverse bacteria are involved in sulfur oxidation in the rice paddy ecosystem than previously considered.

The biological oxidation of hydrogen sulfide to sulfate is one of the main reactions of the global sulfur cycle that occurs in volcanic and other extreme environments (10). The sulfur oxidation reactions in these ecosystems are performed by prokaryotes of the domains Archaea and Bacteria (10). Sulfur-oxidizing bacteria have been isolated from extreme environments, such as hot springs, solfatara fields, and deep-sea hydrothermal vent sites, and characterized for biochemical and ecological analyses in media with high concentrations (20–40 mM) of thiosulfate.

Masuda *et al.* (25) revealed that *Bradyrhizobium diazoefficiens* USDA110 (formerly *Bradyrhizobium japonicum*), a symbiotic nitrogen-fixing bacterium in soil, and several other members of the *Bradyrhizobiaceae* family had the ability to grow chemolithoautotrophically under low concentrations of thiosulfate (0.04–4 mM), which was used as an electron donor (25). The thiosulfate oxidation of *B. diazoefficiens* USDA110 was previously reported to be controlled by the *soxY_1_* gene at *sox* locus I, which is homologous to the sulfur-oxidizing (Sox) system of *Paracoccus pantotrophus* (25). Well-known sulfur-oxidizing bacteria, such as *P. pantotrophus*, have the ability to grow chemoautotrophically with 20 mM thiosulfate (9), while *B. diazoefficiens* USDA110 and other *Bradyrhizobiaceae* members do not (25). These findings suggest that thiosulfate-oxidizing bacteria require thiosulfate to be at an optimum concentration in order to be used as an electron donor. *Rhodopseudomonas palustris*, a phototrophic bacterium, has been shown to produce hydrogen gases using low concentrations of thiosulfate (0.1–6 mM) as an electron donor and light as an energy source (12). Li *et al.* (23) demonstrated that filamentous fungi with *Bradyrhizobium* endosymbionts had the capacity to oxidize sulfur, and suggested that *Bradyrhizobium* may also oxidize thiosulfate. These findings also indicate that unidentified oligotrophic sulfur-oxidizing bacteria with the ability to oxidize at lower concentrations of inorganic sulfur compounds are present in natural environments in which sulfur concentrations are relatively low.

Biological sulfur oxidation has also been proposed to occur in rice paddy fields. It has been detected in the oxidized surface soil layer and rhizosphere, in which oxygen leaks from the aerenchyma system of rice plants (11, 22, 36). Sulfur-oxidizing bacteria may also be abundant at the oxidized surface soil layer and rhizosphere of rice paddy fields. Although total sulfur levels in rice paddy fields vary widely (83–1,176 mg kg^−1^ soil) (22), sulfur concentrations in a rice paddy field were found to be lower than those in a clay fraction of Vitric and Eutric Andosol around a volcano (3,300–14,700 mg kg^−1^ soil) (6).

Some bacterial groups have been isolated as sulfur-oxidizing bacteria from rice paddy fields using enrichment cultures with a high concentration (20 mM) of thiosulfate (30). These bacteria have been considered to oxidize thiosulfate and be involved in biological sulfur oxidation in rice paddy fields. However, such copiotrophic isolation procedures have failed to isolate oligotrophic sulfur-oxidizing bacteria (data not shown). Therefore, other unidentified oligotrophic sulfur-oxidizing bacteria may also exist in rice paddy fields and take part in biological sulfur oxidation.

In the present study, we investigated the responses of bacterial communities in rice-planted pots to sulfur fertilization, no fertilization, CaCO_3_ fertilization, and CaSO_4_ fertilization. The bacterial communities associated with the oxidized surface soil layer and rice roots were analyzed on the basis of the bacterial 16S rRNA genes. We showed that the relative abundance of some bacterial groups was higher in sulfur-fertilized pots than in control pots. Based on comparisons of bacterial communities with and without sulfur amendments, we attempted to identify oligotrophic sulfur-oxidizing bacteria in rice paddies.

## Materials and Methods

### Preparation of soil samples and rice plants

Soil samples (0–20 cm in depth) were collected from an experimental rice paddy field at the Kashimadai Experimental Station (Tohoku University; 38° 27′ 37′ N, 141° 15″ 33′ E, 4 m above sea level) in June 2009. Field soil was sieved with a 0.5-cm mesh and 12 kg of dried soil was used to fill Wagner pots (1/2000 a). Pots were randomly assigned to three treatments (no fertilization, CaCO_3_ fertilization, and CaSO_4_ fertilization) with three replications for each. The CaCO_3_ treatment was included in order to eliminate the effects of Ca in CaSO_4_ fertilizer. Ten grams of CaCO_3_ (2.0 Mg ha^−1^) or 17 g of CaSO_4_·2H_2_O (3.4 Mg ha^−1^) was added to pots in order to achieve an equivalent molar concentration of Ca (0.10 mol), and then mixed thoroughly. Tap water was subsequently added to just below the top of the pot. Rice (*Oryza sativa* L. ‘Nipponbare’) seeds were germinated on filter paper in a petri dish at 30°C. The germinated seeds were transferred to a commercial potting soil mix (Gousei-Baido No. 3, Mitsui-Toatsu Co., Tokyo, Japan) and grown in a greenhouse for 4 weeks under water-logged conditions. Three seedlings were transplanted into each pot in July 2009, one week after fertilization. Rice plants were grown for 8 weeks in a greenhouse under water-logged and natural light conditions and their shoot lengths, tiller numbers, and shoot fresh weights were measured 56 d after transplanting.

### DNA preparation

An oxidized layer with a light brown color developed approximately 3 cm below the soil surface 56 d after transplanting (Fig. S1). After soil samples had been carefully collected from three independent positions in the oxidized surface soil layer in the respective pots, they were mixed into one analytical sample per pot. Slurry soil samples were centrifuged at 13,600 × *g* at 4°C for 5 min in order to discard the supernatant. Approximately 0.5 g of the soil was transferred into a test tube. DNA was extracted using the Fast DNA SPIN Kit for soil (MP Biomedicals, Santa Ana, CA, USA). Rice roots were washed with tap water and carefully sampled from the soil surface and oxidized surface soil layer (Fig. S1). The roots were then homogenized using a mortar and pestle in liquid nitrogen. Microbial DNA was extracted using bead beating in DNA extraction buffer and subsequent purifications as previously described (13).

### Pyrosequencing of 16S ribosomal RNA (rRNA) genes

Bacterial communities were analyzed by a 454 GS FLX Titanium pyrosequence analysis targeting the V2–V3 regions of the 16S rRNA gene using 454 barcode tags (27). Raw pyrosequencing reads were processed using the Quantitative Insights into Microbial Ecology (QIIME) software package (3). The reads were assigned to each sample according to sample-specific barcodes. Low quality reads with a length shorter than 300 bp, an average quality score lower than 25, mismatching primer sequences, or ambiguous bases (denoted by N) were eliminated from the downstream analysis. Forward and reverse primer sequences were removed from quality-filtered reads. The remaining sequences were clustered into operational taxonomic units (OTUs) at the 97% similarity level. The taxonomy of representative OTUs was assigned using the Ribosomal Database (RDP) naive Bayesian classifier (33) with default parameters. A principal-coordinates analysis (PCoA) was performed using weighted UniFrac distances (24). The 454 pyrosequence reads obtained were deposited in the National Center for Biotechnology Information (NCBI) database under accession ID DRA002421. Tukey’s multiple comparison tests and the Student’s *t*-test were used for statistical analyses.

### Chemical analysis

One volume of distilled water or 1M KCl solution was added to the respective air-dried soils, which was measured using a pH meter (F-52S, Horiba, Tokyo, Japan). Approximately 2.0 g of the surface-oxidized soil layer was carefully collected from each pot in order to measure the concentrations of sulfides and thiosulfate. These samples were stored at −80°C until further analyses. Thiosulfate concentrations in oxidized soil were determined using a high-performance liquid chromatograph (HPLC) with a UV detector at a wavelength of 215 nm (intelligent UV/VIS Detector UV-970, Jasco, Tokyo, Japan) (20). Acid-volatile sulfides in oxidized soil fixed with zinc acetate were measured spectrophotometrically using the methylene blue method (19). Soil from the surface-oxidized layer and -reduced layer was collected from each pot and mixed to measure the percentage of total N, C, and S. Soil was air-dried in the greenhouse. Total N, C, and S in the soil of each pot were measured using a gas chromatograph with a thermal conductivity detector (FlashEA 1112, Thermo Electron Corporation, MA, USA). Tukey’s multiple comparison tests were used for statistical analyses.

## Results

### Soil traits and rice growth

The soil pH [H_2_O] and pH [KCl] of control and CaSO_4_-fertilized pots were similar, but were slightly higher (0.4 to 0.6 increases) in CaCO_3_-fertilized pots than in other pots (Table 1). The total amount of C in soil was higher in control pots than in CaCO_3_- and CaSO_4_-fertilized pots (Table 1). The total amount of S in soil was significantly higher in CaSO_4_-fertilized pots than in CaCO_3_-fertilized pots (Table 1). Thiosulfate and sulfide concentrations were slightly higher in CaSO_4_-fertilized pots than in control and CaCO_3_-fertilized pots; however, no significant differences were observed among the different treatments (Table 1).

The shoot lengths, tiller numbers, and shoot fresh weights of rice plants were measured in each pot and are presented in Table 2. No significant differences were observed in plant growth among the different treatments examined, except for the shoot lengths of rice plants grown in the CaCO_3_- and CaCO_4_-fertilized pots. Overall, these results showed that rice growth did not respond significantly to the sulfur regimes employed in this experiment.

### Pyrosequencing of the 16S rRNA gene and PCoA

**Fig. 1:**
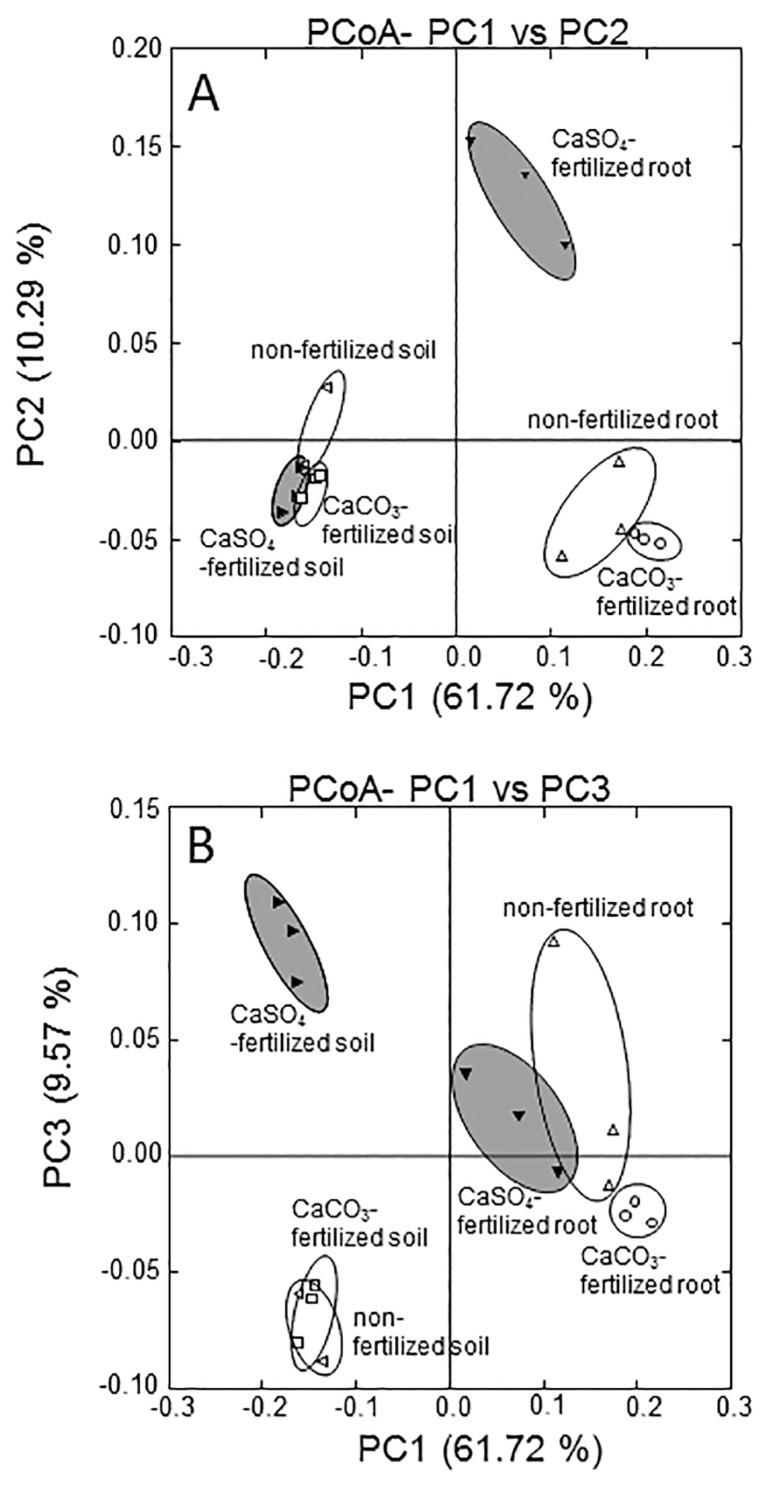
A principal-coordinate analysis for the 16S ribosomal RNA gene of bacteria associated with rice roots and soil in the oxidized surface soil layer in control (no fertilizer), CaCO_3_-fertilized, and CaSO_4_-fertilized pots. When principal component 1 (PC1) and principal component 2 (PC2) were plotted on the *x*- and *y*-axes (A), the bacterial community in CaSO_4_-fertilized root appeared to be separate from those of non-fertilized and CaCO_3_-fertilized roots. When PC1 and principal component 3 (PC3) were plotted on the *x*- and *y*-axes (B), the bacterial community in CaSO_4_-fertilized soil appeared to be separate from those of non-fertilized and CaCO_3_-fertilized soils. Grey indicates the CaSO_4_-fertilized treatment. The ordination was constructed using Unifrac distances. The percentage of variation explained by the plotted principal coordinates is indicated on the axes.

Pyrosequencing of the 16S rRNA gene revealed sulfur fertilizer-dependent changes in bacterial communities. PCoA by Fast UniFrac demonstrated that soil and root microbiomes differentiated along PC1 (61.7%) (Fig. 1A and B). The communities of root-associated bacteria shifted from control and CaCO_3_-fertilized roots to CaSO_4_-fertilized roots along PC2 (10.3%) (Fig. 1A). These results were also supported by the Ribosomal Internal Transcribed Sequence Analysis (RISA) in which CaSO_4_-fertilized root samples differed from control and CaCO_3_-fertilized root samples (Fig. S2, white arrow), whereas the profile of CaCO_3_-fertilized root samples was similar to that of control root samples (Fig. S2). The communities of soil-associated bacteria also shifted from control and CaCO_3_-fertilized soil to CaSO_4_-fertilized soil along PC3 (9.6%) (Fig. 1B). These pyrosequencing results (Fig. 1A and B) suggest that the communities of rice root- and soil-associated bacteria are affected by CaSO_4_ fertilization.

Control and CaCO_3_-fertilized root and soil samples formed a tight cluster separate from CaSO_4_-fertilized samples (Fig. 1A and B). Therefore, the effects of Ca and CaCO_3_ fertilization on root- and soil-associated bacteria at the community level were markedly weaker than those of CaSO_4_ fertilization. These results suggest that the driving force of community shifts due to CaSO_4_ fertilization was most likely explained by bacterial responses to inorganic sulfur oxidation.

### Bacterial communities in rice roots

Pyrosequencing results also revealed changes in the composition of rice root-associated bacteria with the different treatments, particularly CaSO_4_ fertilization (Table 3). The relative abundance of *Acidobacteria*, *Alphaproteobacteria*, *Gammaproteobacteria*, and TM7 was significantly higher in CaSO_4_-fertilized root samples than in control and CaCO_3_-fertilized root samples (Table 3). When the community structure of *Alphaproteobacteria* was examined in detail, *Bradyrhizobiaceae* was found to be significantly more abundant in CaSO_4_-fertilized roots than in control or CaCO_3_-fertilized roots (Table 3). The relative abundance of *Methylocystaceae* was higher in CaSO_4_-fertilized root samples than in control root samples. In contrast, the relative abundance of *Deltaproteobacteria* in the root microbiome was significantly lower in CaSO_4_-fertilized samples than in control and CaCO_3_-fertilized samples (Table 3). These results suggest that sulfur fertilization significantly affects the bacterial composition of rice roots.

### Bacterial communities in soil

The phylogenetic composition based on 16S rRNA gene sequences showed that the relative abundance of TM7 was higher in CaSO_4_-fertilized soil samples than in control and CaCO_3_-fertilized soil samples (Table 3). Conversely, the relative abundance of *Actinobacteria* and *Proteobacteria* was lower in CaSO_4_-fertilized soil samples than in control soil samples (Table 3). The relative abundance of *Rhizobiales* was lower in CaSO_4_-fertilized soil samples than in control soil samples, whereas its relative abundance was significantly higher in the root microbiome than in control and CaCO_3_-fertilized soil samples (Table 3)

## Discussion

In the present study, sulfur fertilization was applied to rice plants grown in flooded pots, and the bacterial communities associated with rice roots and the oxidized surface soil layer were analyzed on the basis of 16S rRNA gene sequences. The PCoA analysis showed that the communities of rice root- and soil-associated bacteria were affected by CaSO_4_ fertilization (Fig. 1A and B). Furthermore, the PCoA analysis and pyrosequencing data suggest that the addition of sulfate mainly induced bacterial community shifts rather than Ca under CaSO_4_ fertilization.

The concentrations of total sulfur, sulfides, and thiosulfate in soil were higher in CaSO_4_-fertilized pots than in control and CaCO_3_-fertilized pots (Table 1). Since thiosulfate is an important intermediate of the sulfur cycle (15), the occurrence of thiosulfate has been interpreted as an indication of ongoing sulfur oxidation and reduction (30, 35). Thiosulfate is readily consumed and generally not detectable in natural environments (30, 35). In the present study, we unequivocally determined the concentration of thiosulfate in all pots using HPLC in order to obtain a sensitive and accurate measurement of thiosulfate in the samples examined.

Thiosulfate concentrations have been reported to be between <10–150 μM and <0.05–2,000 μM in the pore water of planted rice pots and marine sediments, respectively (35, 38). In the present study, the concentration of thiosulfate was low in control [0.227 g kg^−1^ wet soil] and CaSO_4_-fertilized [0.254 g kg^−1^ wet soil] soil samples (Table 1). The soil water content was previously reported to range between 31.2 and 39.7% in rice paddy soil (17). We calculated thiosulfate concentrations in the soil water content on the assumption that the thiosulfate measured in this study was completely dissolved in soil water. Thiosulfate concentrations in the soil water of control and CaSO_4_-fertilized soils were 5.01–6.48 and 5.70–7.26 mM, respectively. The higher concentration of thiosulfate in CaSO_4_-fertilized soil may have been due to the high turnover rates of reduced sulfur compound oxidation. These concentrations of thiosulfate were similar (4 mM) in which *B. diazoefficiens* USDA110 and other *Bradyrhizobiaceae* members can oxidize thiosulfate in a growth experiment (25). The relative abundance of *Bradyrhizobiaceae* increased two-fold under CaSO_4_ fertilization. Many strains of the *Bradyrhizobiaceae* family have been isolated as endophytes of rice roots (4, 32), and a metagenomic analysis showed that they were associated with the rice root (14). These results suggest that *Bradyrhizobiaceae* oxidize thiosulfate and play an important role in inorganic sulfur oxidation in the rice rhizosphere.

The relative abundance of *Methylocystaceae* was higher in CaSO_4_-fertilized root samples, similar to the shift in *Bradyrhizobiaceae* (Table 3). *Methylocystaceae*, including *Methylosinus*, which oxidize methane, have been reported to be abundant in the root tissues of field-grown rice plants (2). Although the whole genomic sequences of *Methylosinus trichosporium* OB3b, an obligate aerobic methane-oxidizing alphaproteobacterium, and *Methylocystis* sp. SC2, an aerobic type II methanotroph, have already been published, their sulfur oxidation activities have not yet been described (5, 29). In the genomes of *M. trichosporium* OB3b and *Methylocystis* sp. SC2, *soxY* and *soxZ* homologous genes were identified by our survey (WP_003614697 and YP_006591467, respectively), and may exhibit the ability to oxidize thiosulfate.

The results of the present study showed that *Acidobacteria*, *Actinobacteria*, *Gammaproteobacteria*, and candidate division TM7 were abundant in CaSO_4_-fertilized root samples (Table 3). Previous studies showed that some members of *Actinobacteria* were grown autotrophically using sulfur as an energy source (26), while some members of *Gammaproteobacteria* that had the Sox system had the ability to oxidize inorganic sulfur compounds in deep-sea hydrothermal fields (37). These findings suggest that *Acidobacteria* and *Gammaproteobacteria* oxidize inorganic sulfur compounds and take part in sulfur oxidation in the rice rhizosphere.

The 16S rRNA gene sequences of *Acidobacteria* have been detected in hot springs (28) and the rice root (31). Furthermore, TM7 16S rRNA gene sequences have been detected in soil (7, 8) and marine sponges (7). An analysis of genomic sequences did not identify any *sox* homologous genes in the published genome of TM7 derived from acetate-amended sediment, activated sludge samples (1, 18), or in *Acidobacteria* isolated from sediments in acidic drainage and clover pasture soil (34). The present study clearly showed that the relative abundance of TM7 and *Acidobacteria* was significantly increased by the application of CaSO_4_ fertilizer. The relative abundance of TM7 was also significantly increased in CaSO_4_-fertilized soil. The relative abundance of bacteria, particularly *Acidobacteria* and *Actinobacteria*, were previously shown to be influenced by changes in soil pH (21). However, the difference observed in soil pH among fertilizer treatments (Table 1) was markedly less than that in the pH profiles of their abundance (21). Thus, soil pH was not a main contributing factor to changes in the relative abundance of *Acidobacteria* and *Actinobacteria* in CaCO_3_-fertilized pots. Thus, we suggest that some bacterial groups of TM7 and *Acidobacteria* grow syntrophically with sulfur-oxidizing bacteria under CaSO_4_ fertilization.

Sulfur-oxidizing bacteria, such as *Beggiatoa* sp., *Thiobacillus* sp., *Xanthobacter* sp., and *Mesorhizobium* sp., have been isolated from the rice rhizosphere and are considered to play important roles in sulfur oxidation in paddy fields (11, 16, 31). However, our results suggest that phylogenetically diverse bacterial groups are involved in the sulfur cycle under paddy field conditions. The isolation of oligotrophic sulfur-oxidizing bacteria in conjunction with their functional and genetic characterization is needed in order to obtain a better understanding of sulfur oxidation in the paddy field ecosystem.

## Supplementary Material



## Figures and Tables

**Table 1:** Soil pH, total concentrations of N, C, and S in soil, and amounts of sulfides and thiosulfate in the wet oxidized surface soil layer of control (no fertilizer), CaCO_3_-fertilized, and CaSO_4_-fertilized pots

Treatment	Soil pH		Total element content (g kg^−1^)			Sulfur species in soil (g kg^−1^ of wet oxidized surface soil)	
		
	H_2_O	KCl	N	C	S	Sulfides	Thiosulfate
Control	4.12 ± 0.01*^a^*	5.24 ± 0.06*^b^*	1.75 ± 0.17*^a^*	19.1 ± 1.0*^a^*	0.37 ± 0.05*^ab^*	5.9 ± 0.4*^a^*	0.227 ± 0.03*^a^*
CaSO_4_-fertilizer	4.15 ± 0.03*^a^*	5.05 ± 0.03*^a^*	1.40 ± 0.12*^a^*	14.5 ± 1.1*^b^*	0.45 ± 0.03*^b^*	9.0 ± 3.0*^a^*	0.254 ± 0.145*^a^*
CaCO_3_-fertilizer	4.57 ± 0.01*^b^*	5.69 ± 0.01*^c^*	1.38 ± 0.20*^a^*	15.0 ± 2.4*^b^*	0.28 ± 0.04*^a^*	6.4 ± 0.5*^a^*	0.156 ± 0.075*^a^*

Different letters indicate significant differences between columns at *P* < 0.05. The values mean the average and standard deviations (*n*=3).

**Table 2:** Growth measurements (shoot length, tiller number, and shoot weight) of rice plants in control (no fertilizer), CaCO_3_-fertilized, and CaSO_4_-fertilized pots

Treatment	Shoot length (cm)	Tiller number	Shoot weight (g)
Control	104 ± 3*^a^*	7.1 ± 1.5*^a^*	31 ± 4*^a^*
CaSO_4_ fertilizer	101 ± 2*^b^*	8.0 ± 0.9*^a^*	29 ± 4*^a^*
CaCO_3_ fertilizer	107 ± 3*^a^*	6.8 ± 0.1*^a^*	29 ± 5*^a^*

Different letters indicate significant differences between columns at *P* < 0.05. The values mean the average and standard deviations (*n*=9).

**Table 3:** Relative abundance of the 16S rRNA gene of soil and rice roots in the oxidized surface soil layer of control (no fertilizer), CaCO_3_-fertilized, and CaSO_4_-fertilized pots

Taxon	Root			Soil		
	
	Control	CaSO_4_	CaCO_3_	Control	CaSO_4_	CaCO_3_
*Acidobacteria*	3.2*^a^*	5.7*^b^*	2.7*^a^*	18.8	12.3	13.5
*Actinobacteria*	4.7	7.5	4.6	8.1*^a^*	3.0*^b^*	5.4*^ab^*
*Chloroflexi*	4.5	2.0	3.4	3.0	5.1	3.9
*Firmicutes*	10.2	7.6	7.7	1.8	0.9	1.8
*Proteobacteria*	46.5	41.6	55.1	35.1*^ab^*	32.0*^b^*	36.3*^a^*
*Alphaproteobacteria*	6.3*^a^*	11.4*^b^*	5.1*^a^*	11.9	8.9	9.8
*Rhizobiales*	4.6*^a^*	7.5*^b^*	3.6*^a^*	7.0*^a^*	4.3*^b^*	5.5*^ab^*
*Bradyrhizobiaceae*	1.2*^a^*	2.4*^b^*	1.1*^a^*	1.2	0.8	1.0
*Methylocystaceae*	1.8	2.5^*^	1.0	1.5	0.4	1.2
*Betaproteobacteria*	12.5	11.7	20.5	10.7*^a^*	7.6*^b^*	11.4*^a^*
*Deltaproteobacteria*	20.7*^a^*	11.7*^b^*	17.7*^a^*	8.0	7.9	9.5
*Gammaproteobacteria*	1.0*^a^*	2.2*^b^*	1.4*^ab^*	3.3	5.4	4.3
TM7	0.5*^a^*	8.8*^b^*	0.3*^a^*	0.2*^a^*	2.8*^b^*	0.4*^a^*

Different letters indicate significant differences between columns at *P*<0.05. The asterisk indicates significance (*P*<0.01), calculated by the Student’s *t*-test between CaSO_4_- and CaCO_3_-fertilized roots. Three replications were prepared, except for control soil (two replications).
