# Supplementary material for: Sulfur Fertilization Changes the Community Structure of Rice Root-, and Soil- Associated Bacteria
Source: Microbes Environ. 2016 Mar 5;31(1):70–5. doi: 10.1264/jsme2.ME15170 (PMC4791119; doi:10.1264/jsme2.ME15170)
Supplement: Supplementary file 1 [file 31_70_s1.pdf]

Supplementary Information for "Sulfur fertilization changes community structure of rice-root- and soil-associated bacteria"

Sachiko Masuda, Zhihua Bao, Takashi Okubo, Kazuhiro Sasaki, Seishi Ikeda, Ryo Shinoda, Mizue Anda, Ryuji Kondo, Yumi Mori and Kiwamu Minamisawa.

Supplementary Methods

Ribosomal intergenic spacer analysis (RISA). The abundance and diversity of bacteria were semi-quantitatively evaluated by bacterial RISA. RISA was performed using bacterial primers ITSF/ITSReub as previously described (1).

References for Supplementary Information

1. Ikeda, S., L. E. E. Rallos, T. Okubo, S. Eda, S. Inaba, H. Mitsui and K. Minamisawa. 2008. Microbial community analysis of field-grown soybeans with different nodulation phenotypes. Appl. Environ. Microbiol. 74:5704–5709.

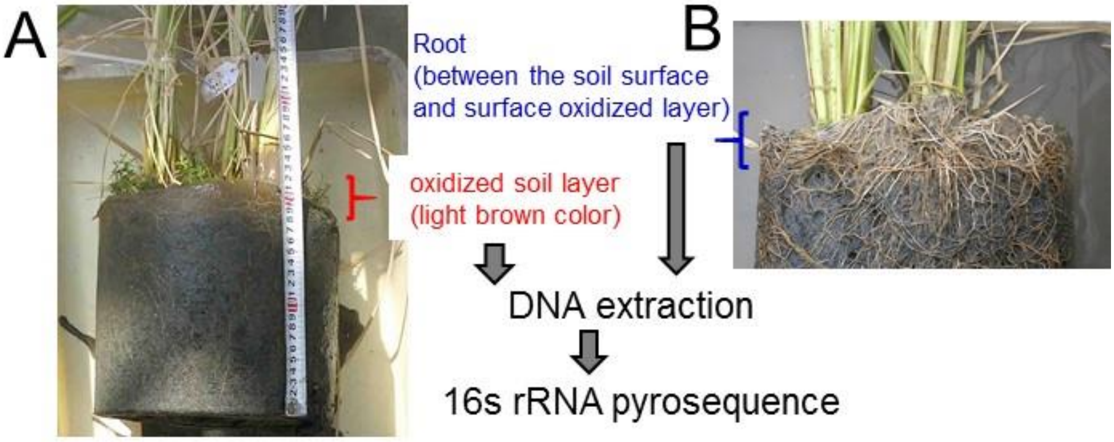

Fig. S1 Soil and root samples in this study. (A) The Wagner pot was carefully removed at 56 days after transplanting. An oxidized soil layer was developed approximately 3 cm below the soil surface. (B) Magnification of oxidized soil layer. The roots were washed and carefully sampled located in the oxidized soil layer.

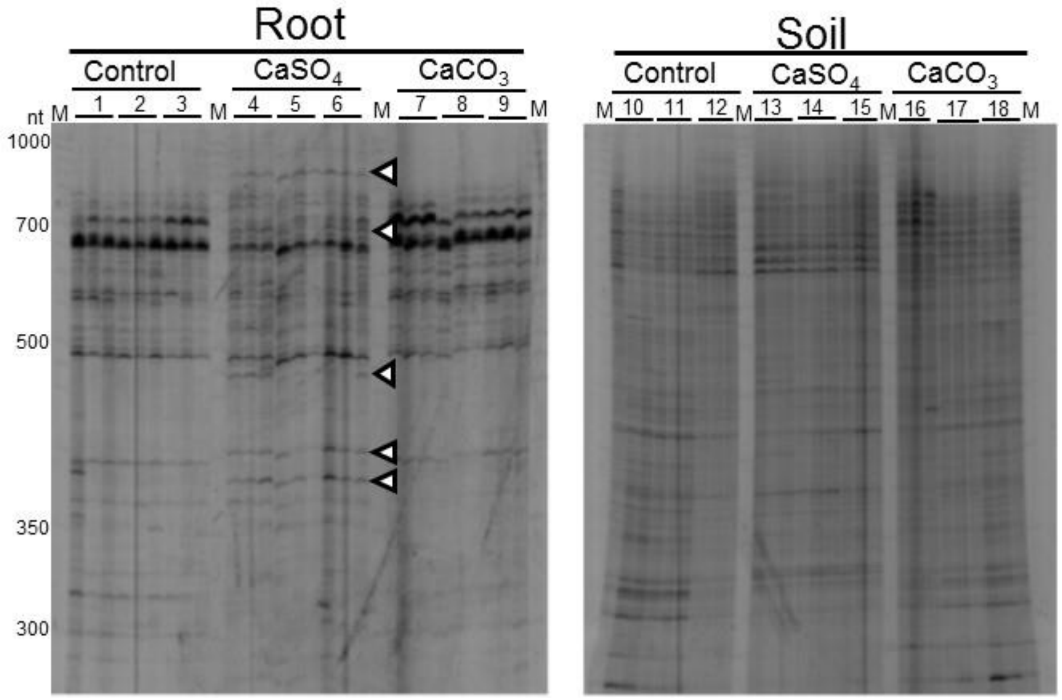

Fig. S2 RISA profile of root- and soil-associated bacteria in control (no fertilizer), $CaCO_3$-fertilized, and $CaSO_4$-fertilized pots. DNA was extracted from soil and roots within the surface oxidized layer. Marked difference in the RISA profile of soil samples was not observed across all treatments. Lane M: MapMarker 1000 (BioVentures, Murfreesboro, TN, USA); Lanes 1–18: PCR products of root- and soil- associated bacteria from different treatments. White arrowheads indicate the specific band of CaSO4-fertilized roots. Each number represents the number of pot samples.
